# DrugFormer: Graph‐Enhanced Language Model to Predict Drug Sensitivity

**DOI:** 10.1002/advs.202405861

**Published:** 2024-08-29

**Authors:** Xiaona Liu, Qing Wang, Minghao Zhou, Yanfei Wang, Xuefeng Wang, Xiaobo Zhou, Qianqian Song

**Affiliations:** ^1^ Center for Computational Systems Medicine McWilliams School of Biomedical Informatics The University of Texas Health Science Center at Houston Houston TX 77030 USA; ^2^ Department of Health Outcomes and Biomedical Informatics College of Medicine University of Florida Gainesville FL 32611 USA; ^3^ Biostatistics and Bioinformatics H. Lee Moffitt Cancer Center and Research Institute Tampa FL USA

**Keywords:** drug resistance, knowledge graph, language model, single‐cell RNA sequencing

## Abstract

Drug resistance poses a crucial challenge in healthcare, with response rates to chemotherapy and targeted therapy remaining low. Individual patient's resistance is exacerbated by the intricate heterogeneity of tumor cells, presenting significant obstacles to effective treatment. To address this challenge, DrugFormer, a novel graph‐augmented large language model designed to predict drug resistance at single‐cell level is proposed. DrugFormer integrates both serialized gene tokens and gene‐based knowledge graphs for the accurate predictions of drug response. After training on comprehensive single‐cell data with drug response information, DrugFormer model presents outperformance, with higher F1, precision, and recall in predicting drug response. Based on the scRNA‐seq data from refractory multiple myeloma (MM) and acute myeloid leukemia (AML) patients, DrugFormer demonstrates high efficacy in identifying resistant cells and uncovering underlying molecular mechanisms. Through pseudotime trajectory analysisunique drug‐resistant cellular states associated with poor patient outcomes are revealed. Furthermore, DrugFormer identifies potential therapeutic targets, such as COX8A, for overcoming drug resistance across different cancer types. In conclusion, DrugFormer represents a significant advancement in the field of drug resistance prediction, offering a powerful tool for unraveling the heterogeneity of cellular response to drugs and guiding personalized treatment strategies.

## Graphical abstract

DrugFormer, a novel graph‐augmented language model, addresses the critical challenge of drug resistance in cancer treatment. By integrating serialized gene tokens and a gene‐based knowledge graph, it provides accurate drug response predictions at the single‐cell level. DrugFormer demonstrates superior performance and identifies resistant cells with potential therapeutic targets, offering a promising solution for personalized cancer therapy.

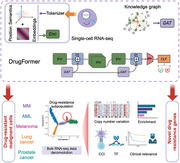

## 1 Introduction

Drug resistance stands as a significant challenge in healthcare,^[^ ^1^ ^]^ underscored by alarming response rates observed in clinical studies. A meta‐analysis of 570 phase II single‐agent clinical trials revealed a median response rate to chemotherapy of merely 11.9%, and this rate is only 30% in the context of personalized targeted therapy.^[^ ^2^ ^]^ Despite ongoing efforts addressing inter‐patient heterogeneity of treatment effects, very limited attention is paid to the intra‐patient heterogeneity resulting in drug resistance. For instance, intra‐tumoral heterogeneity refers to the diversity of cancer cells within a tumor, which can exhibit different genetic, epigenetic, and phenotypic characteristics. This heterogeneity poses challenges for cancer therapy because different subpopulations of cancer cells may respond differently to drugs, leading to treatment tolerance and tumor recurrence. Therefore, it is crucial to interrogate the diversity of drug‐resistant cancer cells and identify specific cellular subpopulations leading to patient‐level resistance.

With the rapid development of technology, single‐cell RNA sequencing (scRNA‐seq) has become a revolutionary technique, providing high resolution for investigating tumor cell resistance at the cellular and cell type levels.^[^ ^3^, ^4^, ^5^, ^6^, ^7^, ^8^, ^9^, ^10^, ^11^, ^12^ ^]^ scRNA‐seq has been applied to exploit drug resistance mechanisms, leading to the discovery of effective targets and the development of optimized therapeutic strategies. For example, Heo et.al. analyzed scRNA‐seq data and identified the cytidine deaminase as the potential druggable target to eliminate resistant cells in lung cancer.^[^ ^13^ ^]^ Li et al. revealed a subpopulation of quiescent stem‐like cells that contributed to the chemoresistance and poor outcomes of AML.^[^ ^14^ ^]^ Those advanced technologies and generated data resources have been collected in extensive datasets, such as the DRMref database,^[^ ^15^ ^]^ which serves as a comprehensive reference map detailing drug resistance mechanisms in human cancer and provides a valuable resource for insightful analyses of drug resistance.

To delve into the drug resistance for individual patients, it is critical to develop tailored methods interrogating how cells in a complex tissue differentially respond to drugs. However, currently, there is a lack of advanced models designed for this purpose. Though several studies, such as DREEP,^[^ ^16^ ^]^ scDEAL,^[^ ^17^ ^]^ SCAD,^[^ ^18^ ^]^ have leveraged existing drug screening databases^[^ ^19^, ^20^ ^]^ to investigate drug response at the single‐cell level, these studies have significant limitations. First, they rely on cell line‐based knowledge as the reference, lacking in vivo properties and failing to mimic real in vivo scenarios, which may result in poor prediction accuracy for cells in tissue samples. Second, their predictions are limited to certain drugs or compounds available in the drug screening databases,^[^ ^19^, ^20^ ^]^ lacking the generalization capability to predict cell responses to real‐world applied drugs. To address those limitations, large language models (LLMs) have emerged as a promising solution for uncovering cellular responses to drugs. Given the existing scRNA‐seq data collected for drug resistance research,^[^ ^15^ ^]^ sophisticated large language models offer a tailored solution that not only aggregates information from extensive in vivo data resources, but also possesses the generalization capability to predict unknown cellular drug response in scRNA‐seq data, facilitating a more comprehensive mechanistic understanding.

Since the BERT model,^[^ ^21^ ^]^ the development of pre‐trained large language models has become more and more successful in natural language processing, as well as in bioinformatics and biomedical areas. For example, OpenAI's GPT^[^ ^22^ ^]^ series and Rostlab's ProtBert^[^ ^23^ ^]^ have achieved great success. Meanwhile, as graphs can represent complex relationships and structures from biological data or external knowledge bases, through incorporating graph structures, graph‐enhanced LLMs can leverage these intricate relationships, leading to a richer and more nuanced understanding of the data. This allows the LLMs to access and utilize graph information or external knowledge more effectively, thus improving their performance. Such graph‐enhanced large language models can be refined to be more accurate, controllable, and adaptable across diverse tasks and application scenarios. In this work, we have proposed our DrugFormer model that leverages the large‐scale drug resistance database^[^ ^15^ ^]^ to achieve accurate predictions of cellular‐level drug resistance. Such a cutting‐edge model is designed with the primary objective of predicting drug resistance and unraveling novel therapeutic targets.

## 2 Results

### 2.1 Overview of DrugFormer

**Figure 1:**
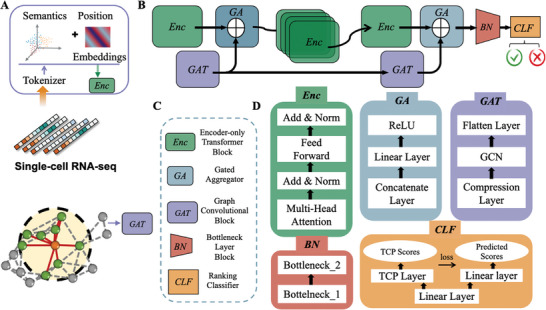
Overview of the DrugFormer model. DrugFormer integrates gene token representations and a gene‐based knowledge graph for drug response prediction. A) Genes are serialized as inputs for the Transformer encoder, while a gene‐based knowledge graph is constructed as input for the graph attention network. B) The overall framework of DrugFormer. C) Explanation of the four types of module components in DrugFormer. D) Detailed structure of each component.

The overall framework of our proposed DrugFormer model is illustrated in **Figure** **1**. DrugFormer integrates both gene representations and a knowledge graph through two processing paths. One path utilizes a Transformer encoder‐based network to extract gene token representations. The other path leverages a knowledge graph utilizing the graph attention mechanism to extract graph information. As shown in Figure 1A, we serialize genes as input for the Transformer encoder. Meanwhile, we construct a gene‐based knowledge graph using haploinsufficiency (i.e., deletion intolerance) and triploidy sensitivity (i.e., duplication intolerance) information. Such gene‐based knowledge graph serves as input for the graph attention network. Figure 1B illustrates the model framework, which consists of four types of modules. Specifically, *Enc* represents the Transformer encoder layer, *GAT* represents the graph attention network layer, *GA* represents the gated aggregation module, and *CLF* represents the output classifier. Figure 1C,D depict the name and specific structure of each network block. In this framework, gene tokens first pass through the Transformer encoder layer to obtain the gene‐based latent representations. The gene‐based graph is processed through the graph attention network to obtain the graph‐based latent representations. Both representations are fused into a combined embedding, which serves as input of the gated aggregation module. Following this, four Transformer encoders are used for deep extraction of the fused embeddings. After an additional layer of graph attention, the fusion embeddings are further enriched with graph information and then serve as input for the output layer for drug response prediction. Through cross‐validation on collected single‐cells from DRMref,^[^ ^15^ ^]^ DrugFormer outperforms other methods. Details are described in the Experimental Section.

### 2.2 Quantitative Evaluation of DrugFormer Performance

**Figure 2:**
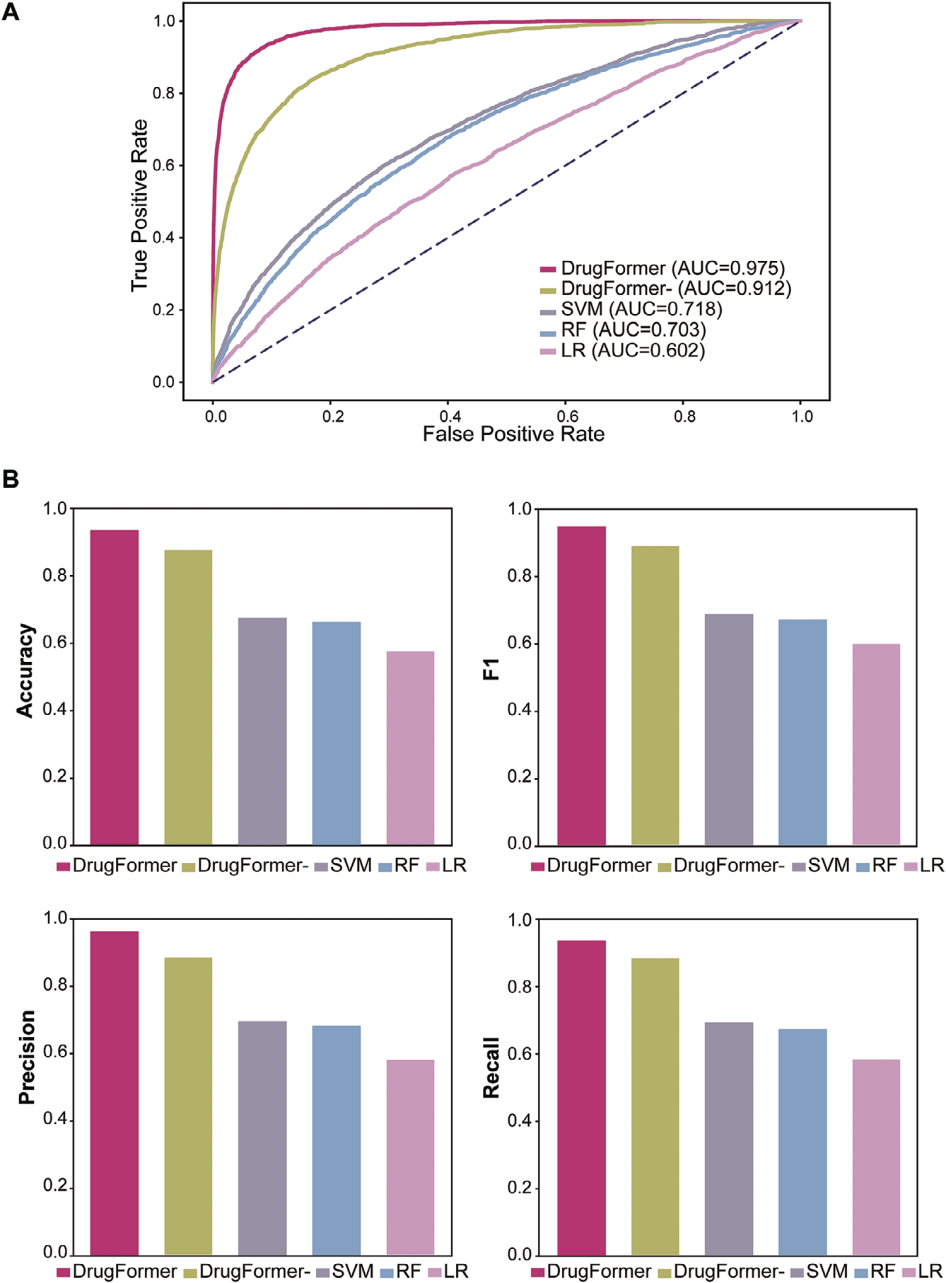
Performance evaluation of DrugFormer. A) AUC of DrugFormer in predicting single‐cell response to drugs based on five‐fold cross‐validation. B) Average values of accuracy, F1, precision, and recall of DrugFormer in predicting cellular drug resistance, based on five‐fold cross‐validation.

Since there were no generalized methods to predict cell responses to real‐world applied drugs, here we included the “DrugFormer‐” model (See Experimental Section) alongside three classic machine learning (ML) models (Random Forest: RF, Support Vector Machine: SVM, and Linear Regression: LR) for comparison. Given the collected single‐cells with drug response labels from DRMref,^[^ ^15^ ^]^ we applied a five‐fold cross‐validation approach, randomly dividing the dataset into five non‐overlapping subsets. For each fold, the remaining four subsets were used to train the model. The performance comparison of these models is illustrated in **Figure** **2**, with all results representing the average metrics from the five‐fold cross‐validation.

As shown in Figure 2A, DrugFormer outperformed other methods in AUC (DrugFormer: 0.975, DrugFormer‐: 0.912, SVM: 0.718, RF: 0.703, LR: 0.602). Notably, in Figure 2B, DrugFormer presented higher accuracy score (acc: 0.932) than DrugFormer‐ (acc: 0.874), SVM (acc: 0.671), RF (acc: 0.659), and LR (acc: 0.573). For other metrics, including F1 score, Precision, and Recall, DrugFormer achieved values of 0.943, 0.958, 0.932, respectively. In contrast, DrugFormer‐ (F1: 0.886, Precision: 0.882, Recall: 0.880), SVM (F1: 0.683, Precision: 0.692, Recall: 0.688), RF (F1: 0.668, Precision: 0.678, Recall: 0.669), and LR (F1: 0.595, Precision: 0.578, Recall: 0.580) presented a poorer performance in these evaluation metrics. Given the lack of existing methods for comparison, we additionally identified two methods, scDEAL^[^ ^17^ ^]^ and SCAD,^[^ ^18^ ^]^ which used cell line data as reference data for predicting drug resistance. Through comparison on their provided datasets (GSE149383 and GSE117872), DrugFormer achieved a significantly higher AUC than both scDEAL and SCAD.

### 2.3 DrugFormer Unveils Resistant Cell State in Multiple Myeloma

To demonstrate the capability of DrugFormer, here we applied it to the scRNA‐seq data profiled from refractory multiple myeloma (MM) patients who were subsequently treated with IMiD drugs.^[^ ^24^ ^]^ Notably, DrugFormer identified the drug‐resistant cells with F1 score as 0.939.

**Figure 3:**
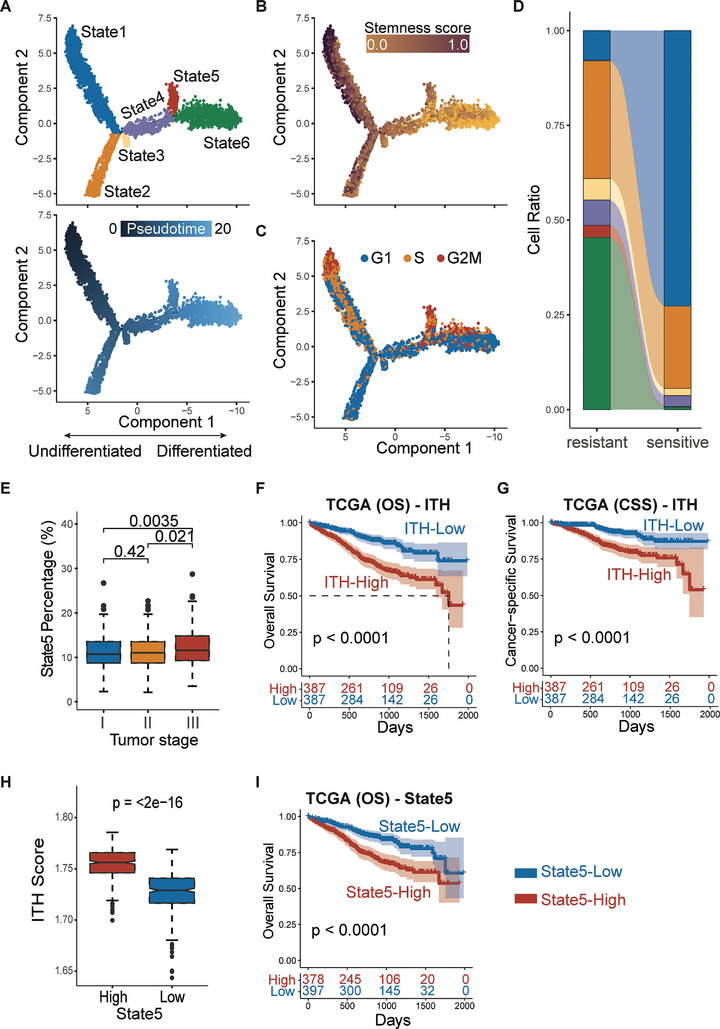
Identification of resistant cell state in multiple myeloma. A) The results of pseudotime analysis by Monocle2. B) The stemness score calculated by CytoTRACE. C) The cell cycle phase predicted by the “CellCycleScoring” function in Seurat. D) Flowchart illustrating the percentage of six states (State1–State5) between the resistant and sensitive cells. E) Boxplot of the percentage of State5 across clinical tumor stages. Data were analyzed with the Wilcoxon Test. There were 261 stage I patients, 270 stage II patients, and 224 stage III patients. The *p*‐values were 0.42, 0.0035, and 0.021 for stage I and stage II, stage I and stage III, and stage II and stage III, respectively. F) Kaplan–Meier curve of overall survival based on ITH level. G) Kaplan–Meier curve of cancer‐specific survival based on ITH‐level. H) The boxplot of the ITH score in patient samples with high and low State5 abundance. Data were analyzed with the Wilcoxon Test. There were 379 State5‐High patients and 397 State5‐Low patients. The *p*‐value was <2e–16. I) Kaplan–Meier curve of overall survival based on State5 abundance. For the Wilcoxon Test and survival analysis, *p* < 0.05 was considered significant.

With DrugFormer recognizing the resistant cells, we next interrogated the cellular dynamics to understand the underlying heterogeneity resulted patient resistance. Herein, we identified the pseudotime trajectory to interpret the cellular states of malignant cells (**Figure** **3**A). For each state on this trajectory, we calculated the stemness score and observed a progressive differentiation potential (Figure 3B). Moreover, based on the cell cycle‐related markers, including 43 genes associated with the S phase and 54 genes associated with the G2M phase, each state is abundant with different cell cycle phases (Figure 3C). Of note, State5 had relatively low stemness scores, but was mostly in the S and G2M phases, representing a subpopulation with low differentiation potential, high proliferation, and increased malignancy. With the resistant cells predicted and confirmed by DrugFormer, next, we calculated the percentage of each state in resistant cells and identified that State5 had a significantly higher percentage in resistant cells (Figure 3D). These results indicated State5 as a unique resistant state in MM.

To further verify the resistant State5, we utilized the MMRF‐COMMPASS patient samples and deconvoluted those samples by the five cell states. Though investigating the relationship between the abundance of each state and the clinical tumor stage, we found that clinical stage III had a significantly higher abundance of State5 (Figure 3E), indicating that State5 was associated with higher malignancy. Using the Shannon entropy, we calculated the intra‐tumoral heterogeneity (ITH) of each patient based on the predicted abundance of each state. Higher ITH was shown to be associated with poorer overall survival and cancer‐specific survival (Figure 3F,G). Importantly, MM patients with high enrichment of State5 tended to have higher ITH (Figure 3H), indicating that the resistant state, i.e., State5, plays a significant role in ITH. Meanwhile, a higher abundance of State5 was observed to be associated with lower overall survival (Figure 3I). These results further demonstrate that State5 is significantly related to patient outcomes and may serve as a potential drug‐resistant subpopulation.

### 2.4 Molecular Mechanisms Underlie Resistant Cell State in Multiple Myeloma

**Figure 4:**
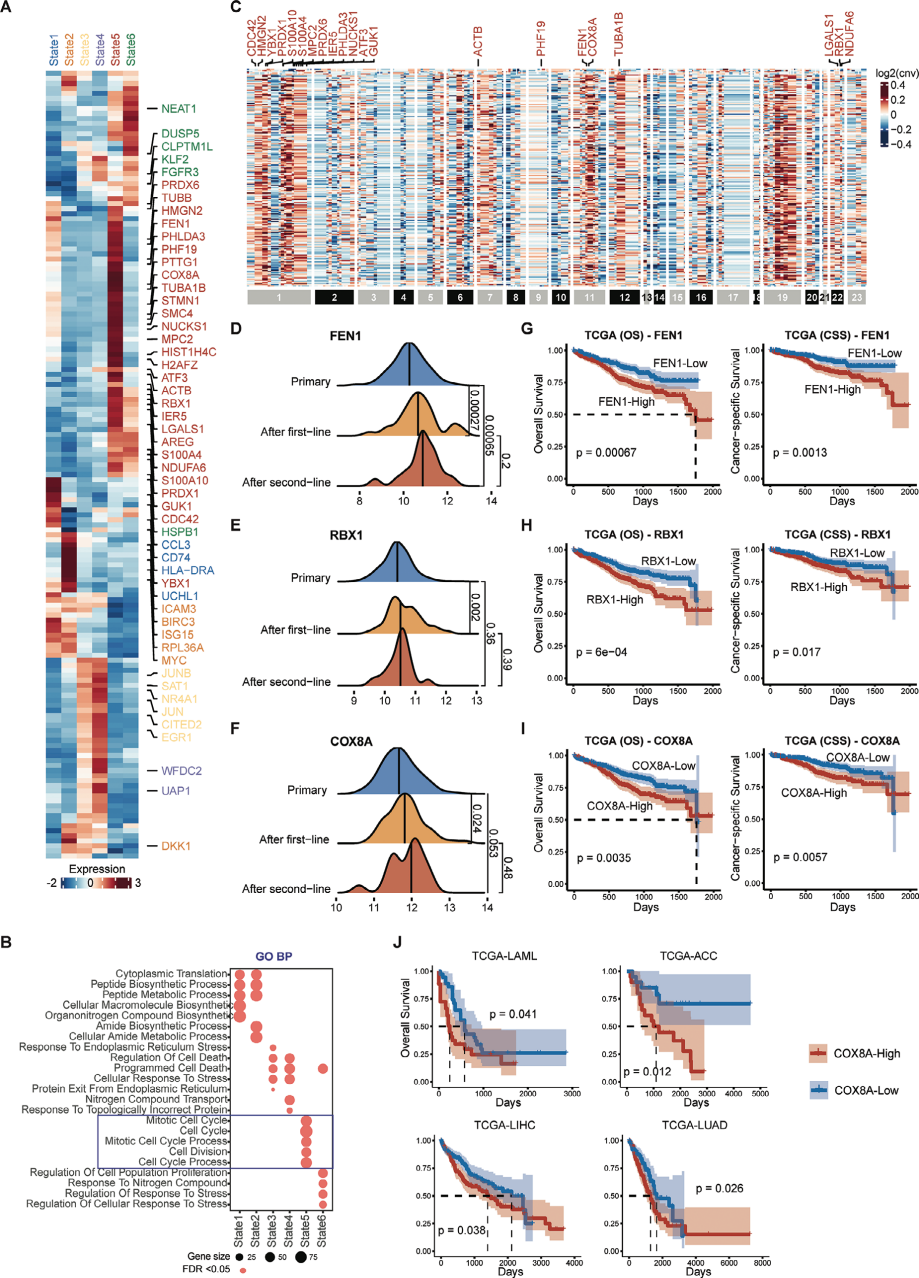
Underlying mechanisms of resistant cell state in MM. A) A portion of the differentially expressed genes in each state. B) GO‐BP enrichment results of differentially expressed genes from each state. FDR < 0.05 was considered significant. C) Inferred copy number variation by copycat software. D) Expression of FEN1 along the treatment line. Data were analyzed with the Wilcoxon Test. There were 776 Primary patients, 64 After first‐line patients, and 19 After second‐line patients. The *p*‐values of FEN1 gene were 0.00027, 0.00065, and 0.2 for Primary and After first‐line, Primary and After second‐line, and After first‐line and After second‐line, respectively. E) Expression of RBX1 along the treatment line. Data were analyzed with the Wilcoxon Test. There were 776 Primary patients, 64 After first‐line patients, and 19 After second‐line patients. The *p*‐values of RBX1 gene were 0.002, 0.36, and 0.39, receptively. F) Expression of COX8A along the treatment line. Data were analyzed with the Wilcoxon Test. *p* < 0.05 was considered significant. There were 776 Primary patients, 64 After first‐line patients, and 19 After second‐line patients. The *p*‐values of COX8A gene were 0.024, 0.053, and 0.48, respectively. G) Kaplan–Meier curve of overall survival and cancer‐specific survival for gene FEN1. H) Kaplan–Meier curve of overall survival and cancer‐specific survival for gene RBX1. I) Kaplan–Meier curve of overall survival and cancer‐specific survival for gene COX8A. J) Kaplan–Meier curve of overall survival based on COX8A expression level in the TCGA‐LAML, TCGA‐ACC, TCGA‐LIHC, and TCGA‐LUAD datasets. For the Wilcoxon Test and survival analysis, *p* < 0.05 was considered significant.

To investigate the underlying genetic mechanisms of resistance in State5, next, we characterized the differentially expressed genes in each cell state especially State5 (**Figure** **4**A). Compared with the other states, State5 highly expressed genes related to the dynamics of cell division (CDC42, YBX1, etc.), DNA repair (FEN1, RBX1, etc.), chromosomal stability (SMC4, etc.), and mitochondrial respiratory and oxidative phosphorylation (COX8A, etc.). Following GO‐BP enrichment analysis, only the upregulated genes of State5 were significantly enriched in processes related to the Mitotic Cell Cycle (Figure 4B). Moreover, with the copy number variations inferred by copycat,^[^ ^25^ ^]^ State5 presented significant copy number amplifications in chromosomes 1, 11, and 19 (Figure 4C). Of note, some upregulated genes of State5, such as FEN1, RBX1, and COX8A,^[^ ^26^, ^27^, ^28^ ^]^ showed copy number amplification. Interestingly, among the MMRF‐COMMPASS patients, the expressions of FEN1, RBX1, and COX8A significantly increased along the patient treatment line (Figure 4D–F). Moreover, the expression of FEN1, RBX1, and COX8A was significantly increased in malignant‐resistant cells compared with malignant‐sensitive cells in the single‐cell dataset (Figure S1, Supporting Information). These results suggest that these three genes were upregulated in drug‐resistant patients. In addition, among the TCGA‐MM patients, high expressions of either FEN1, RBX1, and COX8A were significantly associated with poor overall survival and cancer‐specific survival (Figure 4G–I). Specifically, for COX8A, such association patterns were also observed in some other cancer types of TCGA patients, including ACC, LAML, LIHC, and LUAD (Figure 4J). These findings suggest that COX8A may serve as a potential new target for overcoming drug resistance for several types of cancers.

Next, we further analyzed the cell‐cell interactions and transcriptional regulation of malignant drug‐resistant cells, drug‐sensitive cells, and other cell types in the tumor microenvironment, to delve into the molecular characterization and mechanisms of drug resistance (Figure S2, Supporting Information). Through the cell‐cell interaction analysis (Figure S2A,B, Supporting Information), the results showed that ligand‐receptor pairs (MDK‐NCL and IL16‐CD4) were only present in cell‐cell interactions where sensitive cells served as the source (Figure S2C, Supporting Information). The other ligand‐receptor pair TNFSF13B‐TNFRSF13B was only significantly present in cell‐cell interactions where resistant cells served as the target (Figure S2D, Supporting Information). Previous studies have reported that IL16 induces TME cells migration, which improves cancer therapeutic efficacy.^[^ ^29^ ^]^ An increase in TNFSF13B‐TNFRSF13B could induce the proliferation of malignant cells in multiple myeloma.^[^ ^30^ ^]^ Moreover, transcriptional regulation analysis showed that JUND transcription factors were significantly enriched and up‐regulated in resistant cells (Figure S2E,F, Supporting Information). Previous studies have shown that high expression of JUND is associated with increased malignancy in certain tumors and may promote tumor cell proliferation and invasion.^[^ ^31^ ^]^ These results shed further light on the characterization of drug resistance and the accuracy of our model.

### 2.5 DrugFormer Uncovers Resistant Cell Subpopulation in Different Cancers

To further demonstrate the capability of DrugFormer, we applied it to the scRNA‐seq data profiled from refractory acute myeloid leukemia (AML) patients who were subsequently treated with ficlatuzumab.^[^ ^32^ ^]^ Notably, DrugFormer identified the resistant cells with F1 score as 0.909. With the resistant cells confirmed by DrugFormer, we used similar analyses to interrogate the cellular dynamics to understand the underlying heterogeneity resulted drug resistance in AML.

**Figure 5:**
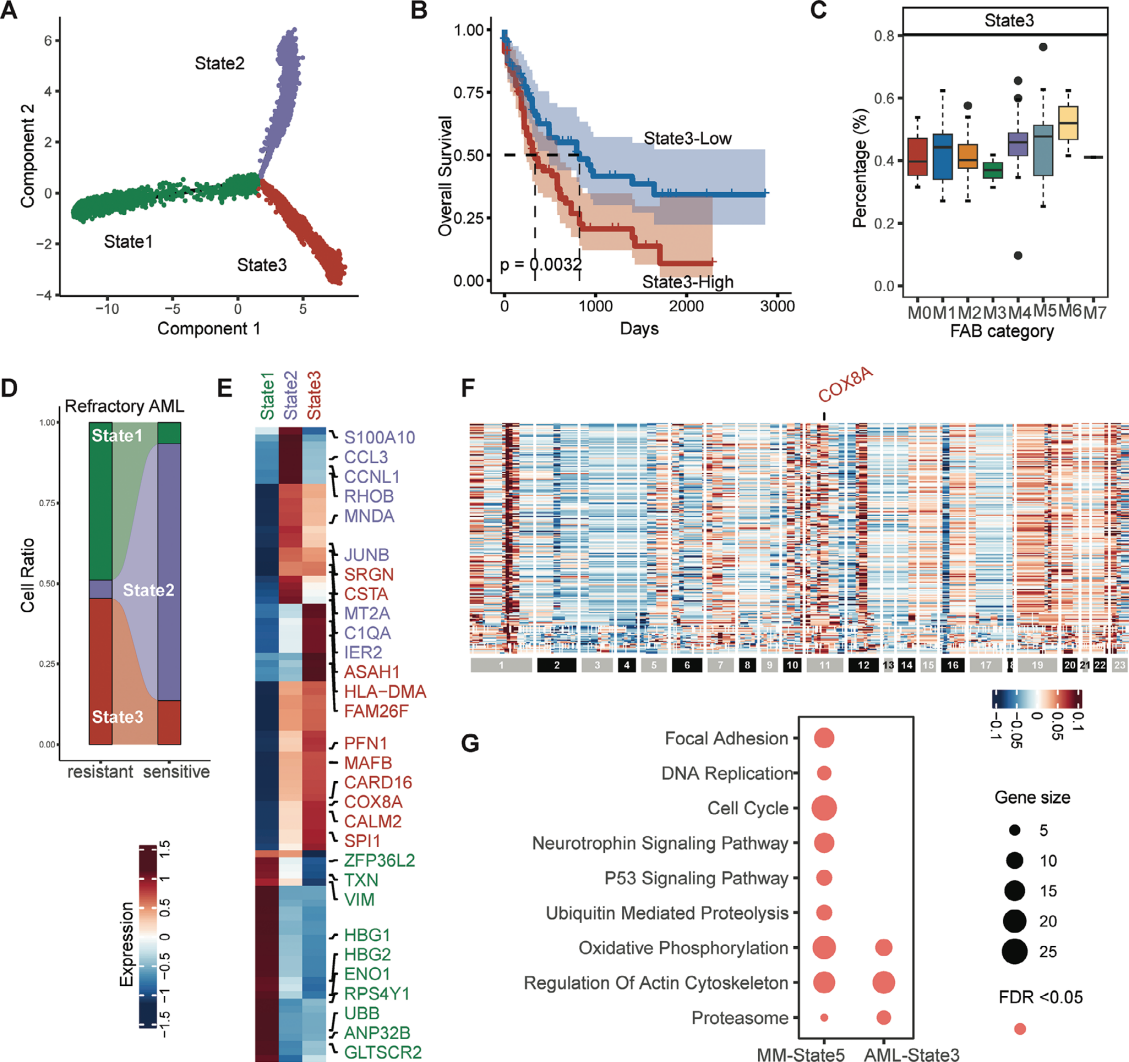
Resistant cell state of acute myeloid leukemia. A) Pseudotime analysis results using Monocle2 for the AML dataset. B) Kaplan–Meier curve of overall survival based on State3 abundance level in the TCGA‐LAML dataset. *p* < 0.05 was considered significant. C) Boxplot of State3 proportions across clinical tumor subtypes in the AML dataset. There were 15 M0 patients, 35 M1 patients, 38 M2 patients, 14 M3 patients, 29 M4 patients, 15 M5 patients, 2 M6 patients, and 1 M7 patients. D) Flowchart illustrating the percentage of three states between the resistant and sensitive cells in the AML dataset. E) Subset of differentially expressed genes in each state of the refractory AML dataset. F) Inferred copy number variation of State3 in the AML dataset. G) KEGG enrichment results of differentially expressed genes from MM‐State5 and AML‐State3. FDR <0.05 was considered significant.

First of all, we inferred the malignant cell trajectories and revealed the cell states from the AML scRNA‐seq data (**Figure** **5**A). Subsequently, we utilized those annotated cell states (State1–State3) as references to deconvolve those cell state proportions in TCGA‐LAML patient samples. Then we analyzed the relationship between the proportion of each cell state and overall survival. The results showed that a higher proportion of State3 was associated with worse survival (Figure 5B). This specific State3 was more abundant in advanced tumors or clinical subtypes composed of mature cells (Figure 5C). Meanwhile, most of the State3 cells in the AML scRNA‐seq dataset came from drug‐resistant patients (Figure 5D), suggesting State3 as the unique drug‐resistant state. Similarly, the upregulated differentially expressed genes (DEGs) of State3 were identified (Figure 5E). Consistent with the findings in the MM dataset, State3 of the Refractory AML also had significant copy number amplification in chromosome 1q and COX8A (Figure 5F). Interestingly, the State3 of AML shares the high enriched pathways including the “oxidative phosphorylation,” “regulation of actin cytoskeleton,” and “Proteasome” pathways with the State5 of the MM dataset (Figure 5G). These results suggest that the specific resistant states from different cancer types may share similar molecular characteristics, enabling the identification of a common potential drug resistance‐specific subpopulation and potential targets, such as COX8A for overcoming drug resistance.

**Figure 6:**
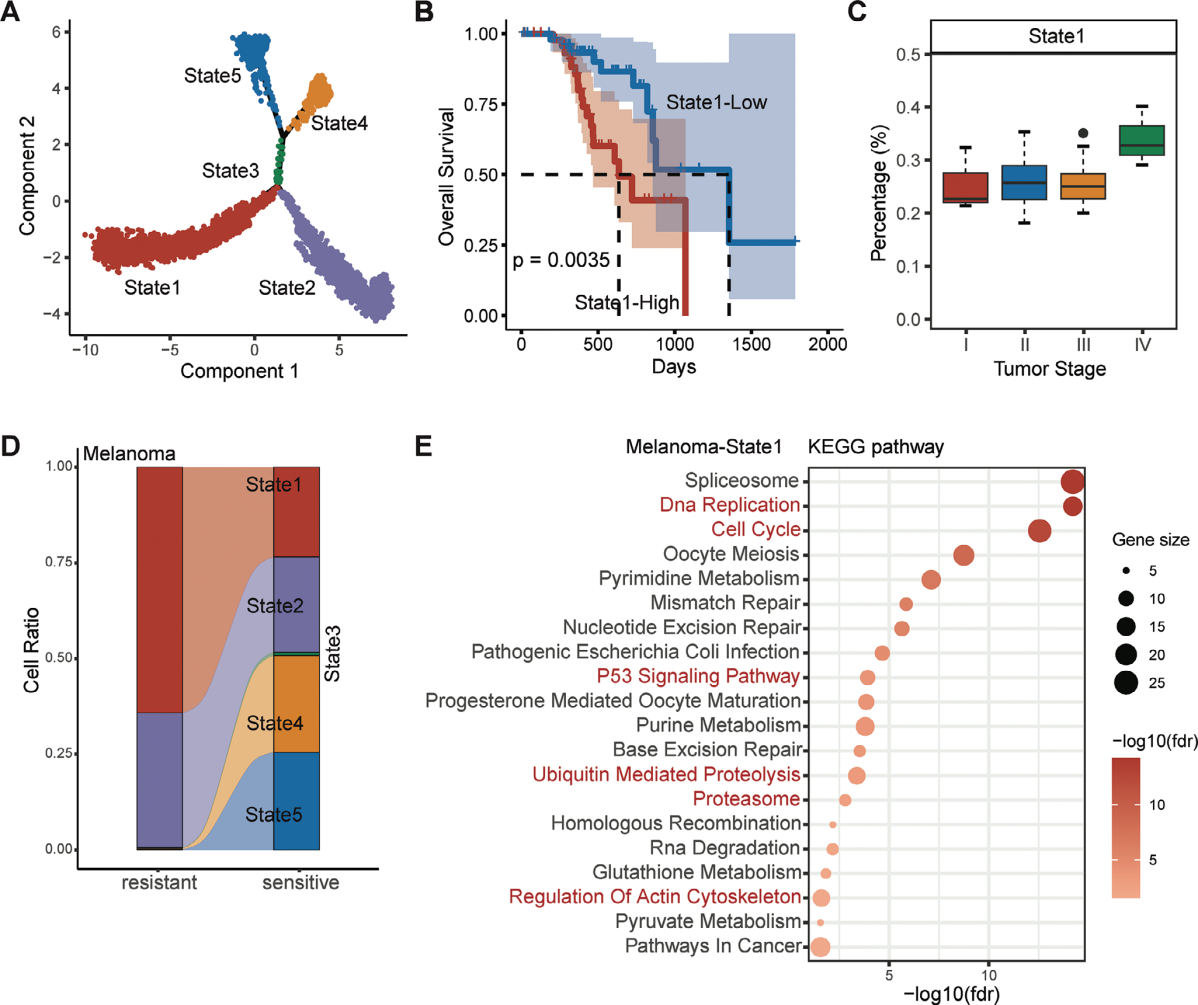
Resistant cell state of melanoma. A) Pseudotime analysis results using Monocle2 for the melanoma dataset. B) Kaplan–Meier curve of overall survival based on State1 abundance level in the TCGA‐SKCM dataset. *p* < 0.05 was considered significant. C) Boxplot of State1 proportions across clinical tumor subtypes in the melanoma dataset. There were 3 stage I patients, 66 stage II patients, 27 stage III patients, and 3 stage IV patients. D) Flowchart illustrating the percentage of three states between the resistant and sensitive cells in the melanoma dataset. E) KEGG enrichment results of differentially expressed genes from melanoma‐State1. FDR <0.05 was considered significant.

Next, we applied DrugFormer to the scRNA‐seq data of solid tumors including melanoma (**Figure** **6**), small cell lung cancer (Figure S3A, Supporting Information) and prostate cancer (Figure S3B, Supporting Information). The melanoma scRNA‐seq dataset has the BRAFV600E mutation, which was subsequently treated with dasatinib.^[^ ^33^ ^]^ The lung cancer dataset was from a small cell lung cancer (SCLC) CDX model, which was subsequently treated with chemotherapy.^[^ ^34^ ^]^ The prostate cancer dataset was profiled before enzalutamide treatment.^[^ ^35^ ^]^ Notably, DrugFormer identified the resistant cells with high F1 scores of 0.951 and 0.896, and 0.876 in these three cancers, respectively (Table S1, Supporting Information). These results demonstrate that our model can be applied to both liquid and solid tumors with strong generalization and reliability.

With the resistant cells identified by DrugFormer, we then interrogated the cellular dynamics to understand the underlying heterogeneity that resulted in drug resistance. For the melanoma scRNA‐seq dataset, we then inferred the malignant cell trajectories and revealed the cell states (**Figure** **6**A). Five cell states (State1–State5) were identified and used to decompose the cell state proportions in TCGA‐SKCM patient samples. Then we analyzed the relationship between the proportion of each cell state and overall survival. The results showed that a higher proportion of State1 was associated with worse survival (Figure 6B). This specific State1 was more abundant in the advanced clinical stage (Figure 6C). Meanwhile, most of the State1 cells in the melanoma scRNA‐seq dataset came from drug‐resistant patients (Figure 6D), suggesting State1 as a unique drug‐resistant state. Moreover, enrichment analysis revealed that State1 had highly enriched pathways including the “cell cycle,” “regulation of actin cytoskeleton,” and “Proteasome” pathways (Figure 6E), which were also observed in the State5 of MM dataset and the State3 of AML dataset (Figure 5G). For the lung cancer scRNA‐seq data, through trajectory analysis, we also found a cell state (State2) in which most of the cells were from drug‐resistant patients (Figure S3A, Supporting Information). For the prostate cancer scRNA‐seq data, most cells in states 5, 6, and 7 of the trajectory were also abundant in drug‐resistant patients (Figure S3B, Supporting Information). Those identified resistant cell states of different cancer types may share similar molecular characteristics, enabling the identification of a common potential drug resistance‐specific subpopulation and potential targets for overcoming drug resistance.

## 3 Discussion

The intratumoral intricate heterogeneity exacerbates individual patient resistance, posing a primary hurdle to the effectiveness of targeted therapies. Therefore, it is critical to delve into how varying cell populations in different regions of tumor lesions manifest resistance or incomplete responses to treatment to enhance the overall efficacy of drug therapy. In this work, we have developed a tailored model, DrugFormer, to interrogate how cells and genes in a complex tissue respond to drugs differentially. DrugFormer harnesses advanced natural language processing capabilities to decipher extensive datasets from the DRMref database.^[^ ^15^ ^]^ The performance of DrugFormer has been validated through comprehensive benchmarking. DrugFormer not only enhances the accuracy of predicting resistance but also sheds light on previously undiscovered targets for therapeutic intervention. Our model represents a significant advancement in the field, holding the potential to redefine how we approach drug resistance prediction and the identification of novel targets, ultimately contributing to more effective and personalized medical treatments.

In the application of DrugFormer to the scRNA‐seq data of multiple myeloma, we unveiled a specific cancer cell state exhibiting stronger drug resistance while presenting elevated expressions of FEN1, RBX1, and COX8A. FEN1 and RBX1 are two genes related to DNA repair, which have been reported to be associated with drug resistance in several cancers.^[^ ^26^, ^27^, ^28^ ^]^ COX8A, or Cytochrome C Oxidase Subunit 8A, is a nuclear‐encoded subunit of cytochrome coxidase (COX), which is the terminal enzyme complex of the mitochondrial respiratory chain. It plays a crucial role in the regulation of mitochondrial oxidative phosphorylation and energy production. The discovery regarding COX8A suggests that it may serve as a potential new target for overcoming drug resistance in MM. Possible mechanisms of drug resistance may involve the increase in chromosome copy number leading to upregulation of COX8A gene expression, thereby enhancing energy support, promoting cancer cell proliferation, and contributing to drug resistance. Previous study shows that COX8A may lead to a number of inherited disorders such as Leigh‐like syndrome and epilepsy. These disorders are often associated with mitochondrial dysfunction, which in turn is closely linked to the development of cancer.^[^ ^36^ ^]^ Another recent work identifies four stemness‐associated genes including COX8A in intrahepatic cholangiocarcinoma (ICC). Functional studies indicate that COX8A is associated with the self‐renewal ability of ICC and transgenic expression of COX8A could enhance chemoresistance of cholangiocarcinoma cells.^[^ ^37^ ^]^ These studies provide further evidence for COX8A as a new target to overcome drug resistance. Since our study focuses on method development, we anticipate validating this target in our future work.

Our findings in MM highlight the intra‐tumoral heterogeneity related to drug tolerance. Such heterogeneity poses challenges for cancer therapy because different subpopulations of cancer cells may respond differently to drugs, leading to treatment tolerance and recurrence. Therefore, it is crucial to use DrugFormer to identify the drug‐resistant cells, thus provide interpretable insights to study drug resistance mechanisms. Given the advantages of DrugFormer, we foresee several aspects for improvement. While the current DrugFormer is specifically designed for single‐cell RNA‐seq data, we envision the development of a spatial omics‐based large language model utilizing emerging spatial datasets. Such an upgrade will unveil the spatial cellular response to drugs, enabling precise patient treatment by identifying drug‐resistant tumor cells within tumor lesions and elucidating the cell‐cell communications underlying such resistance. Furthermore, despite the promising performance of the DrugFormer model, its interpretability may pose a challenge. To address this, the employment of explainable AI approaches^[^ ^38^ ^]^ will help understand the contributions of cells or genes to drug resistance. Additionally, DrugFormer faces the limitations in terms of generalization regarding data collection insufficiency, which may impact the applicability of DrugFormer. If data collection is inadequate, the dataset may not represent the broader population or various conditions. To address this limitation, we are working on collecting more drug treatment‐related single‐cell data and expanding the collection to include diverse populations and sufficient sample sizes, thereby enhancing the generalizability and reliability of DrugFormer.

## 4 Conclusion

DrugFormer represents a significant advancement in addressing the critical challenge of drug resistance in healthcare. By leveraging a novel graph‐augmented language model, DrugFormer integrates serialized gene tokens and a gene‐based knowledge graph to predict drug resistance at the single‐cell level with high accuracy. Comprehensive single‐cell data analysis from different cancer types highlights the efficacy of DrugFormer in identifying resistant cells and uncovering underlying molecular mechanisms. DrugFormer not only enhances the precision of predicting drug response but also reveals unique drug‐resistant cellular states and potential therapeutic targets such as COX8A. This powerful tool offers valuable insights into the heterogeneity of cellular responses to drugs, ultimately guiding personalized treatment strategies and paving the way for more effective cancer therapies.

## 5 Experimental Section

Here, a novel graph‐enhanced language model termed DrugFormer was introduced, which harnessed the Transformer^[^ ^39^ ^]^ and Graph Attention Network^[^ ^40^ ^]^ architectures to predict cell‐level drug response. Figure 1 illustrates the architecture of DrugFormer, which applied the Encoder‐only Transformer Block to incorporate both gene sequence information but also graph information.

### Encoder‐Only Transformer Block

As provided in *Statistical Analysis*, for each gene symbol *g_i_* in the scRNA‐seq data,^[^ ^41^ ^]^ the high‐dimensional embedding of the token sequence was $t_{i}\in R^{256}$ of gene *g_i_*. For each cell in the scRNA‐seq data, the top‐expressed genes (*N*_1_ = 2048) were selected, thus the input of Transformer encoder was $T\;={\left\{t_{i}\right\}}_{i=1}^{N_{1}}$, $t_{i}\in R^{256}$. The knowledge graph was denoted as $G\;={\left\{g_{i}\right\}}_{i=1}^{N_{2}}$, $g_{i}\in R^{k\times k}$, with adjacency matrix $A\in R^{N_{2}\times N_{2}}$. *k* represented the dimension of eigenvalue. *N*_2_ represented the number of genes in the scRNA‐seq data.

DrugFormer used the encoder‐only transformer structure as the backbone. Six‐layer encoder was used and each encoder block consists of an attention layer and a feedforward neural network layer, along with residual connections and normalization operations.^[^ ^42^ ^]^ The attention layer had eight attention heads, with an embedding dimension as 256.

Given the multiple attention heads (*h* = 8), the input to each attention head was annotated as $T_{h}\in R^{N\times 32}$, where 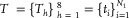. The ouput of *h*‐th attention head *Z_h_* was:

(1) $$\;Z_{h}=\;Attention_{h}=softmax\left(\frac{Q_{h}W_{h}^{T}}{C}\right)V_{h}\;$$

where *Q_h_* = *Linear_q_* (*T_h_*) was the query matrix, *K_h_* = *Linear_k_* (*T_h_* ) was the key matrix, and *V_h_* = *Linear_v_* (*T_h_* ) was the value matrix. *C* was a constant used for scaling. The output of each attention head (*Z_h_*) concatenated as $Z\;=\;\left[Z_{1},.,Z_{8}\right]\in R^{N_{1}\times 256}$ and normalized by layer normalization, i.e.,

(2) $$Z^{\prime }=\;LayerNorm\left(T+Z\right)$$

where $LayerNorm\;\left(\alpha \right)=\left(\alpha -\mu \right)/\sqrt{\sigma^{2}+\varepsilon }$, $\mu \;=\frac{1}{n}\;\sum_{i\;=\;1}^{n}\alpha$, and $\sigma^{2}=\frac{1}{n}\;\sum_{i\;=\;1}^{n}{\left(\alpha -\mu \right)}^{2}$.

Next, a feed‐forward layer for nonlinear transformation and a normalization layer were used, with output as $T^{\left(1\right)}={\left\{t_{i}^{\left(1\right)}\right\}}_{i=1}^{N_{1}}\;,t_{i}^{\left(1\right)}\in R^{256}$, i.e.,

(3) $$T^{\left(1\right)}=\;LayerNorm\left(max\left(0,Z^{\prime }W_{1}+b_{1}\right)W_{2}+b_{2}\right)$$

Here *T* ^(1)^ was the output of the transformer encoder block. The entire model comprised six transformer encoders, each encoder block was denoted as *Enc*(·). The output of *l‐th* transformer encoder was *T* ^(*l*)^, *l* ∈ [1, 6]. Specifically, for the first transformer encoder, *T* ^(1)^ =  *Enc*(*T*).

### Graph Attention Block

To leverage the knowledge graph, the graph attention block with one‐layer graph attention network was used. For two neighboring gene *i* and gene *j* in the graph *G*, this network learned the attention weight *e*_*i*,*j*_ as:

(4) $$e_{i,j}=LeakyReLU\left(a^{⊤}\left[w_{i}^{1}g_{i}∥w_{j}^{1}g_{j}\right]\right)\;$$

where $a^{⊤}\in R^{1\times 512}$ was a learnable vector, $w_{i}^{1}\in R^{256\times k}$ and $w_{j}^{1}\in R^{256\times k}$ were learnable weight matrices. $g_{i}\in R^{k\times 1}$ and $g_{j}\in R^{k\times 1}$ were features of node *i* and node *j*. ∥ represented the concatenation operation, *LeakyReLU*(·) was the activation function, i.e.,

(5) $$LeakyReLU\;\left(\alpha \right)=\left\{\begin{array}{c} \alpha ,\;\;if\;\alpha >0\\ \gamma \cdot \alpha ,\;if\;\alpha \leq 0 \end{array}\right.$$

where α was the input, γ∈R was a small constant used to control the negative slope. When α > 0, *LeakyReLU*(·) was the same as *ReLU*(·); when α ≤ 0, *LeakyReLU*(·) used a small negative slope γ to multiply input α.

The attention coefficient α_*i*,*j*_ was then obtained by normalizing the attention weight *e*_*i*,*j*_ through the *softmax*(·) function, i.e.,

(6) $$\alpha_{i,j}=\frac{\exp\left(e_{i,j}\right)}{\sum_{k\in N\left(i\right)\cup \left\{i\right\}}\exp\left(e_{i,k}\right)}\;$$

where N(i) represented the neighbor nodes of node *i*.

Herein, the aggregated representation $g_{i}^{\prime }$ considering weighted neighbor nodes, i.e., $g_{i}^{\prime }=ReLU\left(\alpha_{i,i}\;w_{i}^{2}g_{i}+\sum_{j\in N(i)}\alpha_{i,j}w_{j}^{2}g_{j}\right)$, where $w_{i}^{2}\in R^{256\times k}$ and $w_{j}^{2}\in R^{256\times k}$ were learnable parameters. Therefore, the node embeddings of the entire graph w $G^{\prime }\;={\left\{g_{i}^{\prime }\right\}}_{i=1}^{N_{2}}\;g_{i}^{\prime }\in R^{256}$. The Graph Attention Block was denoted as *GAT*(·), i.e., *G*′ =  *GAT*(*G*). This node embedding was then aligned with the same list of genes within input *T*, thus we have $\tilde{G}\;={\left\{{\tilde{g}}_{i}\right\}}_{i=1}^{N_{1}}$, where ${\tilde{g}}_{i}\in R^{256}$.

### Gated Aggregator

Following the graph attention block, the embeddings of knowledge graph ($\tilde{G}$) was fused with gene token embeddings (*T* ^(1)^) through the gated aggregator. The gated aggregator enabled the fusion of knowledge graph and gene token using a gating mechanism. In this module, the first layer was designated as a concatenated operation. For each gene *i*, the concatenated operation was shown as:

(7) $$Z_{c}^{\left(1\right)}=\;Concat\left(t_{1}^{\left(1\right)}∥{\tilde{g}}_{1},\text{…},t_{N_{1}}^{\left(1\right)}∥{\tilde{g}}_{N_{1}}\right)$$

where $t_{i}^{\left(1\right)}\in R^{256}$ and ${\tilde{g}}_{1}\in R^{256}$ were denoted above. “∥” referred to concatenating function. $Z_{c}^{\left(1\right)}\in R^{N_{1}\times 512}$ was the concatenated output.

The concatenated features were then projected into a unified embedding space through linear mapping layer and the *ReLU*(·) function, to obtain a complementary embedding representation $Z_{f}^{\left(1\right)}\in R^{N_{1}\times 256}$, i.e.,

(8) $$Z_{f}^{\left(1\right)}=\;ReLU\left(Z_{c}^{\left(1\right)}W_{3}+b_{3}\right)$$

where $W_{3}\in R^{512\times 256}$ and *b*_3_ were the parameters of the linear mapping layer. To this end, the fused embeddings were obtained from the Gated Aggratator *GA*(·), i.e.,

(9) $$Z_{f}^{\left(1\right)}=\;GA\left(T^{\left(1\right)},\;\tilde{G}\right)$$

The fused embeddings $Z_{f}^{\left(1\right)}$ were processed by the second transformer encoder, i.e., $T^{\left(2\right)}=\;Enc\left(Z_{f}^{\left(1\right)}\right)$. The output *T* ^(2)^ then went through three transformer encoders to obtain a deep representation $T^{\left(5\right)}={\left\{t_{i}^{\left(5\right)}\right\}}_{i=1}^{N_{1}}\;t_{i}^{\left(5\right)}\in R^{256}$, i.e., *T* ^(*l*)^ =  *Enc*(*T* ^(*l* − 1)^), *l* ∈ [3, 5]. To further enhance the graph‐represented information, the deep representation *T* ^(5)^ was fused with knowledge graph by the Gated Aggregator *GA*(·), i.e., $T^{\left(6\right)}=\;GA\left(T^{\left(5\right)},\;GAT\left(\tilde{G}\right)\right)$.

### Output Layer

In the output layer, *T* ^(6)^ was first transformed through the shape function that flattens the elements of *T* ^(6)^ to obtain a 1D dense representation. Then a bottleneck layer was used to compress the flattened information through linear transformation and obtain a sparse representation $Z_{s}\in R^{1\times 32}$,

(10) $$Z_{s}=\;Bottleneck\left(Shape\left(T^{\left(6\right)}\right)W_{4}+b_{4}\right)$$

*Z_s_* further went through the classification layer to get the prediction result *P_T_* of the model. The classification layer was shown as follows:

(11) $$P_{T}=\;sigmoid\left(Z_{s}W_{5}+b_{5}\right)$$

herein, the probability value *P_T_* was obtained within [0,1], which referred to the drug response probability of cell.

### Loss Function

To training the model, the batch size was set to 64 and trained on a single Nvidia A100X‐40C GPU with 40GB memory. The training used five‐folds cross‐validation, and the number of training epochs was 5. The training objective was to minimize the cross‐entropy loss *L*:

(12) $$L\;=\;-\left(y\log\left(P_{T}\right)+\left(1-y\right)\log\left(1-P_{T}\right)\right)$$

where *y* was the ground truth label represented by a one‐hot vector. Specifically,^[^ ^10^ ^]^ represented one class (drug‐resistant cell), and [0, 1] represented the other class (drug‐sensitive cell). The first element of *P_T_* referred to the predicted probability of cell resistance, and the second element was the predicted probability of cell sensitivity.

### Statistical Analysis—Pre‐Processing of Data

Each gene symbol *g_i_* in the scRNA‐seq data was first tokenzied^[^ ^41^ ^]^ as token sequence. Each token sequence was performed with high‐dimensional embedding (both semantic and position embedding). As genes with copy number changes often associate with drug response,^[^ ^43^, ^44^, ^45^ ^]^ the probabilities of gene haploinsufficiency (pHaplo) and triplosensitivity (pTriplo) were collected from Ryan et.al.^[^ ^46^ ^]^ The input gene‐level knowledge graph was constructed based on the similarity of these probability scores. If two gene nodes have similar probability scores, these two genes were connected in the knowledge graph. The node attributes were the gene eigenvalue from Ryan et.al,^[^ ^46^ ^]^ complementing the graph information. For downstream analysis, the downloaded scRNA‐seq dataset was preprocessed by the “SCTransform” function of the Seurat package, including filtering, removal of batch effects and normalization.

### Statistical Analysis—Sample Size for Each Statistical Analysis

For the Wilcoxon‐Test of MM‐State5 percentage between tumor stages, there were 261 stage I patients, 270 stage II patients, and 224 stage III patients. For the Wilcoxon‐Test of the ITH score between High‐ and Low‐ MM‐State5 percentage, there were 379 State5‐High patients and 397 State5‐Low patients. For the Wilcoxon‐Test of the expression of genes (FEN1, RBX1, and COX8A) between different treatment lines of MM, there were 776 Primary patients, 64 After first‐line patients, and 19 After second‐line patients. For the comparison of AML‐State3 percentage between AML FAB subtypes, there were 15 M0 patients, 35 M1 patients, 38 M2 patients, 14 M3 patients, 29 M4 patients, 15 M5 patients, 2 M6 patients, and 1 M7 patients. For the comparison of melanoma‐State1 percentage between tumor stages, there were 3 stage I patients, 66 stage II patients, 27 stage III patients, and 3 stage IV patients. In the Wilcoxon‐Test test for FEN1, RBX1, and COX8A gene expression between malignancy‐resistant and malignancy‐sensitive cells, there were 354 malignant‐resistant cells and 5134 malignant‐sensitive cells.

### Statistical Analysis—Data Presentation

To evaluate the performance of DrugFormer, it was compared with the “DrugFormer‐” model and three classic machine learning (ML) models, i.e., Random Forest (RF), Support Vector Machine (SVM), Linear Regression (LR). Here the “DrugFormer‐” model referred to the ablated DrugFormer framework without leveraging gene‐based knowledge graph, i.e., only six transformer encoder blocks. Quantitative metrics including accuracy, F1 score, AUC, precision, and recall were used to evaluate the benchmarking performance. In Figure 2B, the results of the comparison methods were shown by the average values of the evaluation metrics.

### Statistical Analysis—Statistical Methods

For the *evaluation of proliferation and stemness* for each single‐cell, the signature score of a set of cell cycle‐related markers was computed to assess the cell cycle state of each individual cell. This cell cycle‐related marker set included 43 genes associated with the S phase and 54 genes associated with the G2M phase. Additionally, the stemness score for each single‐cell was computed to evaluate stemness based on transcriptional diversity. For the *Characterization of the differentiation trajectory of malignant cells*, the cellular states were identified based on the pseudotime analysis. For the *Identification of specifically expressed genes and enriched pathways in each cellular state*, genes specifically expressed in each cellular state were identified. Genes with log2 (fold‐change) greater than 0.25 and expressed at least 25% of cells were identified as specific genes of each cellular state. Furthermore, enrichment analysis of KEGG and GO BP pathways was conducted. For the *Deconvolution of bulk transcriptomic data*, the cellular states abundances were estimated of bulk transcriptomic data from MMRF‐COMMPASS, TCGA‐LUAD, and TCGA‐LAML datasets. Single‐cells were used as a reference and labeled as different cellular states. Based on the estimated percentage of different cellular states, the relative abundances of each cellular state were compared in patients with various FAB subtypes and tumor stages. Additionally, Shannon entropy was used to calculate the intra‐tumoral heterogeneity (ITH) of each patient from bulk RNA datasets. For the *Copy number variation analysis*, the copy number variations^[^ ^25^ ^]^ were inferred to reveal underlying genetics mechanisms. This process was conducted for each patient to avoid batch effect. For the evaluation of drug‐resistant genes, overexpressed genes in resistant cells (log2FC >0.25 & p.adjust <0.05) with copy number variation >0.02 were used. For *Survival Analysis*, Kaplan–Meier survival analysis was conducted, and the log‐rank test was used to evaluate the survival differences between groups. For *cell–cell interaction analysis*, significant ligand–receptor interaction pairs were selected with a significant value of *p < 0.05*. The number of interactions between different cell types and the intercellular communication weights were represented by circle plots. Additionally, dot plots were used to show the significant ligand‐receptor pairs in malignant‐resistant cells and malignant‐sensitive cells, serving as source cells and target cells respectively, communicate with other cell types.

### Statistical Analysis—Software for Statistical Analysis

All statistical analyses were conducted in R (version 4.2.3). For the *evaluation of proliferation and stemness* for each single‐cell, the “CellCycleScoring” function within the Seurat package was utilized.^[^ ^47^ ^]^ Additionally, the stemness score was computed for each single‐cell using CytoTRACE,^[^ ^48^ ^]^ a well‐established computational framework for evaluating stemness based on transcriptional diversity. For the *Characterization of the differentiation trajectory of malignant cells*, the R package Monocle2 (version 2.28.0)was used to characterize the differentiation trajectory.^[^ ^49^ ^]^ For the *Identification of specifically expressed genes and enriched pathways in each cellular state*, the “FindMarkers” function was used in the Seurat package. Furthermore, enrichment analysis of KEGG and GO BP pathways was conducted using hypeR (version 1.14.0) package.^[^ ^50^ ^]^ For *Deconvolution of bulk transcriptomic data*, the CIBERSORTx^[^ ^51^ ^]^ tool was used. Due to the input size limitation of CIBERSORTx, single‐cells were randomly selected as input. For each cellular state, half were randomly selected if the number of cells was less than 1500, and one‐third for the others. For the *Copy number variation analysis*, copykat package in R was used to infer copy number variations.^[^ ^25^ ^]^ For *Survival Analysis*, the “ggsurvplot” function in the R package survminer was used. For *cell–cell interaction analysis*, CellChat^[^ ^52^ ^]^ (version 1.5.0) was used. For *transcriptional regulation analysis*, the pySCENIC package was used to identify key transcription factors (TFs) in the different cell types according to a “three‐step” TF–target regulatory network construction^[^ ^53^ ^]^ (version 0.12.1). All parameters were set as default values. Dot plots and heatmap were used to show enriched TFs with significantly upregulated expression in each cell type.

## Conflict of Interest

The authors declare no conflict of interest.

## Author Contributions

X.L. and Q.W. are co‐first authors and contributed equally to this work. X.L. performed data curation, formal analysis, and visualization. Q.W. performed methodology, validation, and software. X.L., Q.W., X.Z, and Q.S. wrote, reviewed, and edited the original draft. M.Z. and Y.W. reviewed and edited the original draft. X.Z. and Q.S. performed funding acquisition. X.Z. and Q.S. performed conceptualization, funding acquisition, project administration, resources, and supervision.

## Supporting information

Supporting Information

## Data Availability

Data sharing is not applicable to this article as no new data were created or analyzed in this study.
