# Supplementary material for: DrugFormer: Graph‐Enhanced Language Model to Predict Drug Sensitivity
Source: Adv Sci (Weinh). 2024 Aug 29;11(40):2405861. doi: 10.1002/advs.202405861 (PMC11516065; doi:10.1002/advs.202405861)
Supplement: Supplementary file 1 — Supporting Information [file ADVS-11-2405861-s001.docx]

Supporting Information

DrugFormer: Graph-enhanced Language Model to Predict Drug Sensitivity

Xiaona Liu^1,†^, Qing Wang^2,†^, Minghao Zhou^2^, Yanfei Wang^2^, Xiaobo Zhou^1,*^, and Qianqian Song^2,*^

^1^Center for Computational Systems Medicine, McWilliams School of Biomedical Informatics, The University of Texas Health Science Center at Houston, Houston, TX 77030, USA.

^2^Department of Health Outcomes and Biomedical Informatics, College of Medicine, University of Florida, Gainesville, FL 32611, USA.

^†^ The authors contributed equally to this work.

*Corresponding authors: Xiaobo Zhou: Xiaobo.Zhou@uth.tmc.edu; Qianqian Song: qsong1@ufl.edu


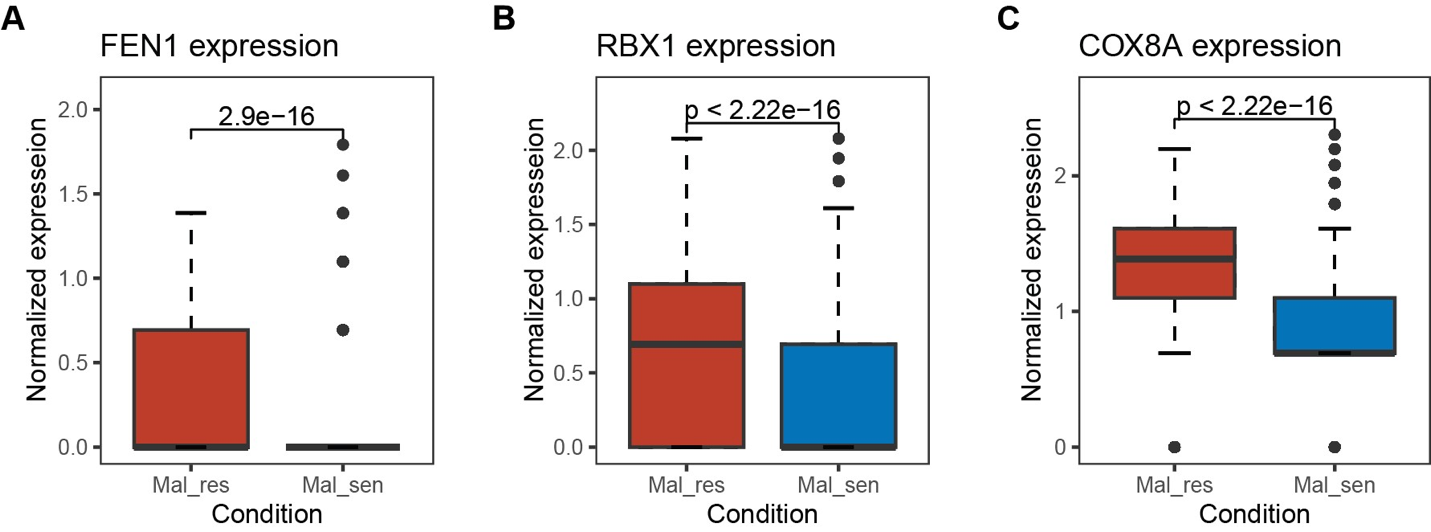


**Figure S1** The relationship between the expression of FEN1, RBX1 and COX8A genes and drug resistance. A) Boxplot of the FEN1 expression in MM malignant-resistant cells and malignant-sensitive cells. P-value was 2.9e-16. B) Boxplot of the RBX1 expression in MM malignant-resistant cells and malignant-sensitive cells. P-value was less than 2.22e-16. C) Boxplot of the RBX1 expression in MM malignant-resistant cells and malignant-sensitive cells. P-value was less than 2.22e-16. There were 354 malignant-resistant cells and 5134 malignant-sensitive cells. Data were analyzed with the Wilcoxon-Test. *P < 0.05* was considered significant.


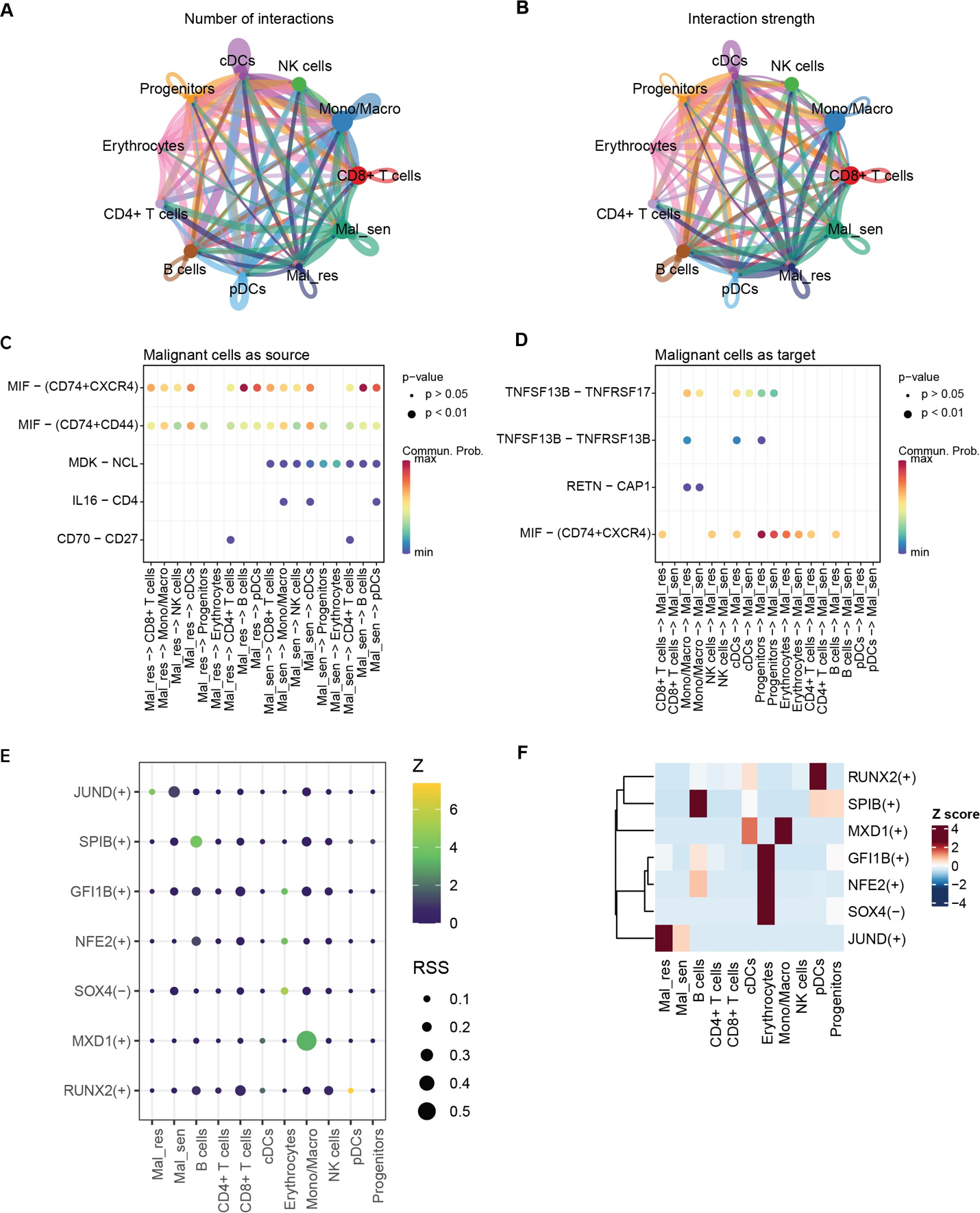


**Figure S2** Cell-cell interactions and transcriptional regulation in different cell types. (A, B) Number of interactions and interaction strength between malignant drug-resistant cells, malignant-sensitive cells, and tumor microenvironmental (TME) cell. (C) Significant ligand-receptor pairs between malignant cells and other TME cells, in which malignant cells served as the source. *P < 0.05* was considered significant. (D) Significant ligand-receptor pairs between malignant cells and other TME cells, in which malignant cells served as the target. *P < 0.05* was considered significant. (E, F) Dot plots and heatmap were used to show enriched TFs with significantly upregulated expression in each cell type.


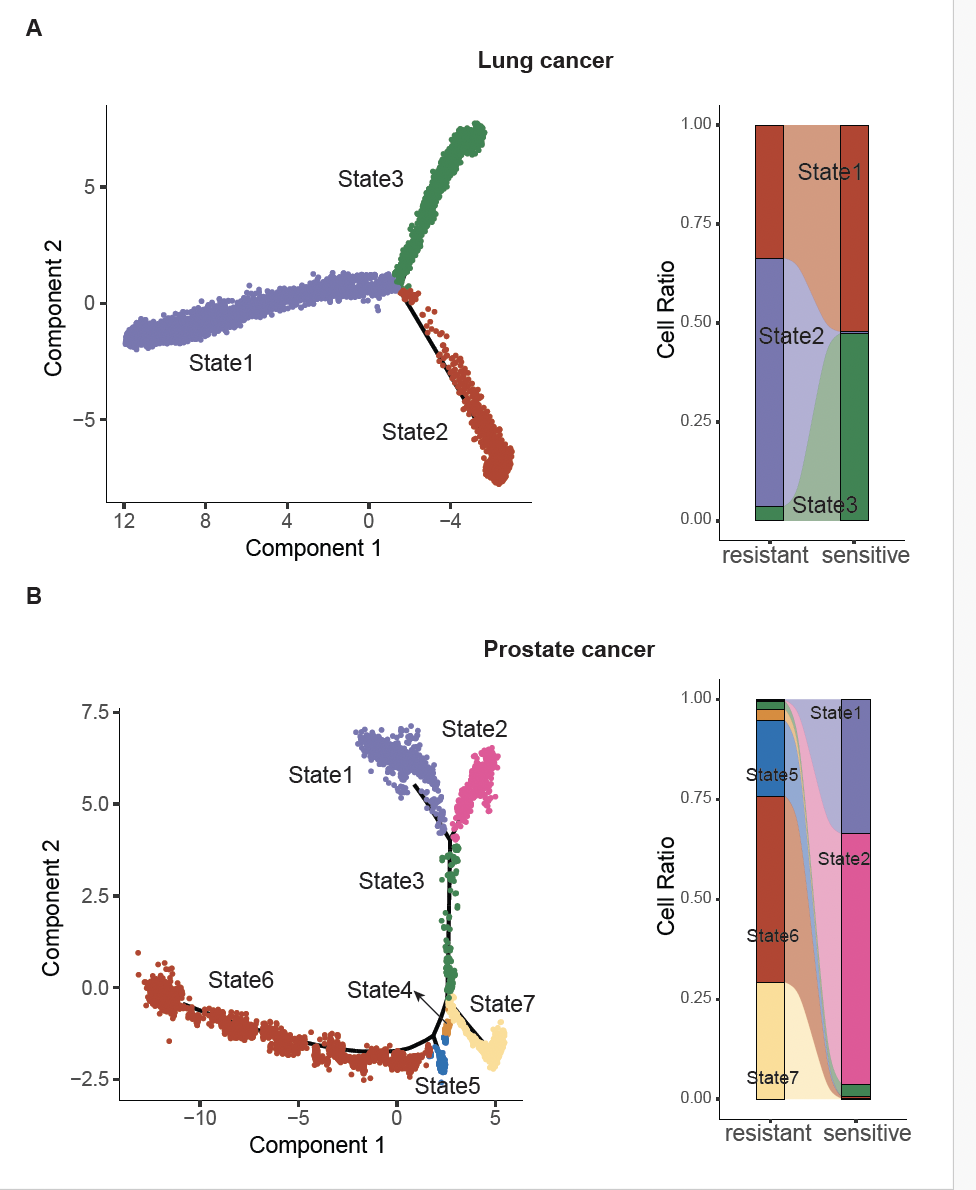


**Figure S3** Resistant cell state of lung cancer and prostate cancer. A) Left panel: Pseudotime analysis results using Monocle2 for the lung cancer dataset. Right panel: Flowchart illustrating the percentage of three states between the resistant and sensitive cells in the lung cancer dataset. B) Left panel: Pseudotime analysis results using Monocle2 for the prostate cancer dataset. Right panel: Flowchart illustrating the percentage of three states between the resistant and sensitive cells in the prostate cancer dataset.

| **Table S1. Performance of DrugFormer, DrugFormer-, SVM, RF, and LR on PC, Mel, and SCLC datasets** | | | | | | |
| --- | --- | --- | --- | --- | --- | --- |
| **Cancer type** | **Accuracy** | **AUROC** | **Precision** | **Recall** | **F1** | **Method** |
| **Prostate cancer (PC)** | 0.932 | 0.966 | 0.906 | 0.847 | 0.876 | DrugFormer |
| **BRAFV600E-mutant melanoma (BR)** | 0.948 | 0.988 | 0.941 | 0.962 | 0.951 | DrugFormer |
| **Small cell lung cancer (SCLC)** | 0.870 | 0.923 | 0.896 | 0.897 | 0.896 | DrugFormer |
| **Prostate cancer (PC)** | 0.843 | 0.890 | 0.846 | 0.739 | 0.788 | DrugFormer- |
| **BRAFV600E-mutant melanoma (BR)** | 0.799 | 0.885 | 0.797 | 0.895 | 0.843 | DrugFormer- |
| **Small cell lung cancer (SCLC)** | 0.740 | 0.767 | 0.732 | 0.743 | 0.737 | DrugFormer- |
| **Prostate cancer (PC)** | 0.659 | 0.709 | 0.658 | 0.640 | 0.649 | SVM |
| **BRAFV600E-mutant melanoma (BR)** | 0.712 | 0.777 | 0.721 | 0.731 | 0.726 | SVM |
| **Small cell lung cancer (SCLC)** | 0.649 | 0.691 | 0.684 | 0.672 | 0.678 | SVM |
| **Prostate cancer (PC)** | 0.610 | 0.687 | 0.650 | 0.621 | 0.635 | RF |
| **BRAFV600E-mutant melanoma (BR)** | 0.689 | 0.724 | 0.700 | 0.712 | 0.706 | RF |
| **Small cell lung cancer (SCLC)** | 0.607 | 0.673 | 0.641 | 0.637 | 0.639 | RF |
| **Prostate cancer (PC)** | 0.557 | 0.632 | 0.611 | 0.601 | 0.606 | LR |
| **BRAFV600E-mutant melanoma (BR)** | 0.638 | 0.689 | 0.674 | 0.666 | 0.670 | LR |
| **Small cell lung cancer (SCLC)** | 0.561 | 0.642 | 0.616 | 0.612 | 0.614 | LR |
